# Low 2016/17 season vaccine effectiveness against hospitalised influenza A(H3N2) among elderly: awareness warranted for 2017/18 season

**DOI:** 10.2807/1560-7917.ES.2017.22.41.17-00645

**Published:** 2017-10-12

**Authors:** Marc Rondy, Alin Gherasim, Itziar Casado, Odile Launay, Caterina Rizzo, Daniela Pitigoi, Aukse Mickiene, Sierk D Marbus, Ausenda Machado, Ritva K Syrjänen, Iva Pem-Novose, Judith Krisztina Horváth, Amparo Larrauri, Jesús Castilla, Philippe Vanhems, Valeria Alfonsi, Alina E Ivanciuc, Monika Kuliese, Rianne van Gageldonk-Lafeber, Veronica Gomez, Niina Ikonen, Zvjezdana Lovric, Annamária Ferenczi, I-MOVE+ hospital working group, Alain Moren

**Affiliations:** 1 EpiConcept, Paris, France; 2 National Centre of Epidemiology, Institute of Health Carlos III, Madrid, Spain; 3 CIBER Epidemiología y Salud Pública, Institute of Health Carlos III, Madrid, Spain; 4 Instituto de Salud Pública de Navarra, IdiSNA, Pamplona, Spain; 5 Inserm, F-CRIN, Innovative clinical research network in vaccinology (I-REIVAC), CIC 1417, Paris, France; 6 Université Paris Descartes, Sorbonne Paris Cité, APHP, CIC Cochin-Pasteur, Paris, France; 7 Istituto Superiore di Sanità, Rome, Italy; 8 University of Medicine and Pharmacy Carol Davila, National Institute for Research Cantacuzino, Bucharest, Romania; 9 Department of Infectious diseases of Lithuanian University of Health Sciences, Kaunas, Lithuania; 10 Centre for Epidemiology and surveillance of infectious diseases, Centre for Infectious disease control, National Institute for Public Health and the Environment (RIVM), Bilthoven, The Netherlands; 11 Epidemiology Research Unit, Epidemiology Department, National Health Institute Doutor Ricardo Jorge, Lisbon, Portugal; 12 Impact Assessment Unit, National Institute for Health and Welfare, Tampere, Finland; 13 Epidemiology Service, Croatian Institute of Public Health, Zagreb, Croatia; 14 Office of the Chief Medical Officer, Budapest, Hungary; 15 Infection Control and Epidemiology Unit, Hôpital Edouard Herriot, Hospices Civils de Lyon, Lyon, France; 16 Emerging Pathogens Laboratory - Fondation Mérieux, Centre International de Recherche en Infectiologie, INSERM U1111, Centre National de la Recherche Scientifique (CNRS), UMR5308, Ecole Nationale Supérieure (ENS) de Lyon, Université Claude Bernard Lyon 1, Lyon, France;; 17 National Institute for Research Cantacuzino, Bucharest, Romania, Faculty of Biology, Bucharest University; 18 Unit of Expert Microbiology, National Institute for Health and Welfare, Helsinki, Finland; 19 The members of the I-Move+ hospital working group are listed at the end of the article

**Keywords:** Influenza, cases control, hospitalisation, vaccine effectiveness, elderly, A(H3N2)

## Abstract

In a multicentre European hospital study we measured influenza vaccine effectiveness (IVE) against A(H3N2) in 2016/17. Adjusted IVE was 17% (95% confidence interval (CI): 1 to 31) overall; 25% (95% CI: 2 to 43) among 65–79-year-olds and 13% (95% CI: −15 to 30) among those ≥ 80 years. As the A(H3N2) vaccine component has not changed for 2017/18, physicians and public health experts should be aware that IVE could be low where A(H3N2) viruses predominate.

In 2016/17, the influenza season in Europe was characterised by an early start (week 46, 2016) and a predominance of A(H3N2) viruses. Overall, 89% of strains reported to the European Centre for Disease Prevention and Control (ECDC) were A(H3N2) viruses [1]. High hospitalisation rates and case fatality ratios were reported among persons aged 65 years and above [2]. The I-MOVE + (Integrated Monitoring of Vaccines in Europe plus) hospital network early estimates, suggested a low 2016/17 seasonal influenza vaccine effectiveness (IVE) against hospitalisation with influenza A(H3N2) among persons aged 65 years and above in the European Union (EU) [3].

Since the A(H3N2) vaccine component has not changed in 2017/18, we present the final 2016/17 season IVE against hospitalisation with influenza A(H3N2) among persons aged 65 years and above in Europe, to inform on the level of IVE that can be expected against A(H3N2) in the upcoming 2017/18 season.

## Study design

We conducted a multicentre hospital-based test-negative design (TND) case-control study in 27 hospitals from 10 countries (Croatia, Finland, France, Hungary, Italy, Lithuania, the Netherlands, Portugal, Romania and Spain) according to a generic protocol adapted to each local setting [4]. The detailed methods are described elsewhere [5]. In brief, hospital teams identified and swabbed patients aged 65 years and above, hospitalised with signs compatible with a severe acute respiratory infection (SARI) defined as at least one systemic and one respiratory sign or symptom. Swabs were tested with reverse-transcriptase polymerase chain reaction (RT-PCR) for influenza A(H3N2), A(H1N1)pdm09 and B. We compared the odds of vaccination between patients positive for influenza A(H3N2) virus and those negative for any influenza virus. We calculated IVE as (1-odds ratio (OR)).

We measured IVE stratified by age group (65–79 year-olds and ≥ 80 year-olds), presence of underlying conditions (diabetes mellitus, cancer, heart or lung disease, and presence of at least two underlying chronic diseases) and 2015/16 seasonal influenza vaccination status. In a one-stage approach, using logistic regression with the study site as a fixed effect, we adjusted IVE estimates for date of symptoms onset, age (as cubic splines) and individual underlying conditions. Using patients unvaccinated in both 2015/16 and 2016/17 seasons as a reference, we computed the effectiveness of being vaccinated in 2015/16 season only, in 2016/17 season only and in both seasons.

## Vaccine effectiveness against influenza A(H3N2) in 2016/17

We included 1,073 influenza A(H3N2) cases, nine A(H1N1)pdm09 cases, 13 cases of influenza B and 1,541 controls between week 47, 2016 and week 14, 2017. Due to the small number of cases, we were not able to measure IVE against influenza A(H1N1)pdm09 and B. We excluded these 22 records from all analyses.

The median age of A(H3N2) cases was 81 years (range: 65−102 years) while that of controls was 80 (range: 65−102 years). Ninety-four percent of cases and 95% of controls had at least one underlying condition (p = 0.14). Controls were more likely than cases to have underlying lung disease (44 vs 37%, p < 0.05), rheumatologic disease (22 vs 15%, p < 0.05) and cancer (24 vs 19%, p < 0.05), to have been hospitalised in the past 12 months (44 vs 33%, p < 0.05) and to be current smokers (26 vs 20%, p < 0.05) (Table 1).

**Table 1:** Characteristics of influenza A(H3N2) hospitalised cases (n = 1,073) and test-negative controls (n = 1,541), I-MOVE + study, Europe, influenza season 2016/17

	Influenza A(H3N2) cases (n = 1,073)		Controls (n = 1,541)	
Median age in years (range)	81 (65–102)		80 (65–102)	
**Characteristic**	**n/N^a^**	**%**	**n/N^a^**	**%**
Aged 65–69 years	457/1,073	42.6	770/1,541	50.0
Sex = male	516/1,072	48.1	815/1,535	53.1
2016/17 seasonal influenza vaccination	556/1,073	51.8	894/1,541	58.0
2015/16 seasonal influenza vaccination	578/1,054	54.8	896/1,525	58.8
**Current and previous vaccination status**				
2016/17 seasonal vaccine only	46/1,054	4.4	99/1,525	6.5
2015/16 seasonal vaccine only	73/1,054	6.9	112/1,525	7.3
2015/16 and 2016/17 seasonal vaccines	505/1,054	47.9	784/1,525	51.4
**Type of 2016/17 vaccine**				
Not vaccinated	517/1,007	48.2	647/1,421	42.0
Inactivated subunit egg	243/1,007	22.6	431/1,421	28.0
Inactivated split virion egg	229/1,007	21.3	321/1,421	20.8
Adjuvanted	18/1,007	1.7	22/1,421	1.4
**Underlying conditions**				
Diabetes mellitus	325/1,072	30.3	473/1,540	30.7
Heart disease	710/1,070	66.4	1,032/1,541	67.0
Lung disease	392/1,069	36.7	672/1,534	43.8
Cancer	201/1,069	18.8	369/1,533	24.1
Renal disease	223/1,071	20.8	319/1,539	20.7
Stroke	125/879	14.2	176/1,287	13.7
Rheumatologic disease	157/1,070	14.7	341/1,539	22.2
Obesity^b^	124/1,062	11.7	154/1,527	10.1
Any underlying condition	996/1,063	93.7	1,456/1,531	95.1
At least two underlying conditions	776/1,025	75.7	1,206/1,491	80.9
Functional impairment	399/1,066	37.4	588/1,529	38.5
Hospitalisations in past 12 months	353/1,063	33.2	668/1,526	43.8
Current smoker	182/901	20.2	318/1,220	26.1
**Potential for misclassification**				
Antivirals received before swabbing	177/1,069	16.0	90/1,535	5.8
Swabbing within 3 days of symptom onset	653/1,073	58.7	876/1,541	56.2
**Study sites**				
Croatia	31/1,073	2.9	13/1,541	0.8
Finland	20/1,073	1.9	50/1,541	3.2
France	119/1,073	11.1	209/1,541	13.6
Hungary	8/1,073	0.7	19/1,541	1.2
Italy	73/1,073	6.8	136/1,541	8.8
Lithuania	67/1,073	6.2	58/1,541	3.8
Navarre, Spain	242/1,073	22.6	290/1,541	18.8
The Netherlands	40/1,073	3.7	63/1,541	4.1
Portugal	49/1,073	4.6	29/1,541	1.9
Romania	90/1,073	8.4	103/1,541	6.7
Spain^c^	334/1,073	31.1	571/1,541	37.1

I MOVE+: Integrated Monitoring of Vaccines in Europe plus.

^a^ N represents the total number of cases or controls with available information.

^b^ Defined as body mass index ≥ 30 kg/m^2^.

^c^ Excluding Navarre.

The one-stage pooled adjusted IVE was 17% (95% confidence interval (CI): 1 to 31) overall; 25% (95% CI: 2 to 43) among patients aged 65–79 years and 10% (95 %CI: −15 to 30) among those aged 80 years and above. Among patients with specific underlying conditions, IVE ranged between 19% (95% CI: −1 to 35) among patients with heart disease and 35% (95% CI: 14 to 51) among patients with lung disease (Table 2).

**Table 2:** Seasonal influenza vaccine effectiveness against influenza influenza A(H3N2) overall and stratified by patient characteristics, I-MOVE + study, Europe, influenza season 2016/17

Population and patient characteristics	Vaccinated /cases	%	Vaccinated /controls	%	Adjusted IVE	95% CI
Aged 65 years and above - age/time^a^	556/1,073	52	894/1,541	58	17	1 to 31
Aged 65 years and above - full model^a^	544/1,041	52	868/1,494	58	14	−3 to 29
Aged 65–79 years - age/time^a^	175/457	38	382/770	50	25	2 to 43
Aged 80 years and above - age/time^a^	381/616	62	512/771	66	13	−12 to 32
**According to underlying diseases**						
Diabetes mellitus	183/320	57	295/468	63	22	−8 to 44
Heart disease	378/703	54	622/1,024	61	19	−1 to 35
Lung disease	209/386	54	440/668	66	35	14 to 51
Cancer	105/198	53	227/362	63	21	−19 to 47
At least two underlying chronic diseases	414/767	54	732/1,196	61	17	−2 to 33
**According to previous vaccination**						
Not vaccinated in 2015/16	46/473	10	99/623	16	39	−3 to 59
Vaccinated in 2015/16	502/572	88	776/887	87	−2	−44 to 28
**Sensitivity analyses**						
Swabbed within 3 days	502/872	58	333/629	53	8	−16 to 28
No antivirals before swabbing	867/1,446	60	509/904	56	14	−3 to 29

I MOVE+: Integrated Monitoring of Vaccines in Europe plus.

^a^ Variables used for adjustment:

-age/time: adjusted for study site, age and onset date (modelled as a restricted cubic spline with 3 and 4 knots respectively);

-full model: adjusted for study site, onset date, age (modelled as a restricted cubic spline with 3 and 4 knots respectively), lung diseases, heart diseases, diabetes, obesity, renal diseases, cancer and hospitalisation in the past 12 months;

-other estimates were adjusted for study site, onset date, age (modelled as a restricted cubic spline with 3 and 4 knots respectively) and hospitalisation in the past 12 months.

The 2016/17 seasonal IVE was −2% (95% CI: −44 to 28) among patients who had received 2015/16 seasonal influenza vaccine and 39% (95 %CI: −3 to 59) among patients not vaccinated in 2015/16 (Table 2). Taking as a reference patients unvaccinated in 2015/16 and 2016/17, IVE for those vaccinated in 2015/16 only was 19% (95% CI: −15 to 42) and IVE when vaccinated both in 2015/16 and 2016/17 was 15% (95 %CI: −3 to 30) (Table 3).

**Table 3:** Seasonal influenza vaccine effectiveness against influenza A(H3N2) by vaccine uptake in 2015/16, 2016/17 and in both seasons, I-MOVE + study, Europe, influenza season 2016/17

Vaccine uptake in 2015/16 and 2016/17 influenza seasons	Cases	Controls	IVE	95% CI
Not vaccinated	427	524	Reference	
2016/17 only	46	99	38	9 to 58
2015/16 only	70	111	19	−15 to 42
Vaccinated in both seasons	502	776	15	−3 to 30

CI: confidence interval; I-MOVE+: Integrated Monitoring of Vaccines in Europe plus; IVE: influenza vaccine effectiveness.

^a^ Adjusted for study site, age and onset date (modelled as a restricted cubic spline with 3 and 4 knots, respectively).

The A(H3N2) vaccine components were A/Switzerland/2013 (3C.3a) in the 2015/16 seasonal vaccine and A/Hong Kong/2014 (3c.2a) in the 2016/17 seasonal vaccine.

## Discussion

In the 2016/17 influenza season, A(H3N2) viruses largely predominated. IVE against hospitalisation with influenza A(H3N2) virus infection among persons aged 65 years and above was low at 17%. The IVE point estimate was even lower (10%) among patients aged 80 years and above. IVE was similar among patients with heart disease, diabetes mellitus and cancer. The IVE point estimate was higher among patients with lung disease. While 95% CIs were largely overlapping, the 2016/17 IVE point estimate was lower (IVE: −2%) among patients vaccinated also in 2015/16 than among those unvaccinated in 2015/16 (IVE: 39%).

Low IVE against influenza A(H3N2) among persons aged 65 years and above has been previously observed in hospital settings [6-8]. A recent meta-analysis measured that the pooled IVE against hospitalisation with influenza A(H3N2) in seasons when circulating and vaccine strains were antigenically different was 14% (95% CI: −3 to 30) among persons aged 65 years and above [9]. It was 43% (95% CI: 33 to 53) in seasons when circulating and vaccine A(H3N2) strains were antigenically similar; 48% (95% CI: 37 to 59) against influenza A(H1N1)pdm09 and 38% (95% CI: 25 to 53) against influenza B [9].

Based on specimens received from week 40/2016 to week 5/2017, available antigenic data from the World Health Organization (WHO) European Region indicated that most circulating viruses that could be analysed were considered as antigenically similar to the 2016/17 vaccine component [10]. Consequently, European data supported the WHO recommendation to maintain the same vaccine component A/Hong Kong/4801/2014 (clade 3C.2a) for influenza A(H3N2) in the 2017/18 season vaccine for the northern hemisphere [11]. However, one third of viruses isolated during the above-mentioned period could not be assigned to an antigenic reporting category, reflecting technical challenges or antigenic changes in circulating viruses. Genetic data from Europe centralised at the ECDC suggested that circulating A(H3N2) viruses had undergone considerable genetic diversification during the above-mentioned period, with the emergence of subclusters within clade 3C.2a and subclade 3C.2a1 [10].

In September 2017, WHO updated the A(H3N2) component to A/Singapore/INFIMH-16-0019/2016 (subclade 3C.2a1) in the 2018 seasonal vaccine for the southern hemisphere [12]. The latest WHO update on 2 October 2017, reported that influenza A(H3N2) viruses were still predominating worldwide in September 2017. Further genetic information was not provided at this stage [13].

Our results taking patients unvaccinated in both 2015/16 and 2016/17 seasons as a reference suggested that influenza vaccination in 2015/16 and/or 2016/17 reduced the risk of influenza-associated hospitalisation among vaccinated patients. Our stratified results suggested that 2015/16 vaccination modified the 2016/17 IVE. Although too imprecise to be conclusive, our results could suggest that patients vaccinated in both seasons benefited from a residual protection from the 2015/16 vaccine, with no additional effect of the 2016/17 vaccine uptake.

## Conclusion

Our results suggest a low IVE against hospitalised influenza A(H3N2) among persons aged 65 years and above, particularly among patients aged 80 years and above. They also suggest a modifying effect of 2015/16 influenza vaccination on 2016/17 IVE. The A(H3N2) virus component included in the 2017/18 vaccine will remain the same as in the 2016/17 season. The latest WHO influenza surveillance report suggests that influenza A(H3N2) viruses were predominating worldwide in August 2017. Low IVE may be expected during the 2017/18 season in case of predominant circulation of A(H3N2) viruses. However, IVE against influenza A(H1N1)pdm09 and B are usually reported to be higher. Close monitoring of virological surveillance data will be required to prompt early promotion of complementary measures such as the use of antivirals or non-pharmaceutical interventions.

## Notes

**Members of the I-Move + hospital working group:** EpiConcept: Marta Valenciano Spain: F. Pozo (National Centre for Microbiology, National Influenza Reference Laboratory, WHO-National Influenza Centre, Institute of Health Carlos III), M. García, M. Latorre (Dirección General de Salud Pública, Aragón), M. Omeñaca (H.U. Miguel Servet, Aragón), M. Oribe Amores, N. Muñoz (Subdirección de Salud Pública Gipuzkoa, País Vasco), G. Cilla (H. U. Donostia, Pais Vasco) Navarre: L Fernandino, I Martínez-Baz (Instituto de Salud Pública de Navarra, Pamplona), A Navascués, A Pérez-García, A Aguinaga, C Ezpeleta (Complejo Hospitalario de Navarra, IdiSNA, Pamplona, Spain) Italy: A. Bella, Eva Charlotte Appelgren, M.R. Castrucci, S. Puzelli (Istituto Superiore di Sanità, Rome), M.Chironna, C. Germinario (Policlicnico Hospital, University of Bari); F. Ansaldi, A. Manini, E. Montomoli (Department of Molecular and Developmental Medicine, University of Siena) Romania: E. Lupulescu, M. Lazar, M.E. Mihai, C.M Cherciu, S. Dinu, C. Tecu (National Institute for Research Cantacuzino), M. Nitescu, R. Bacruban, D. Azamfire, A. Dumitrescu, E. Ianosik. (INBI Prof Dr Matei Bals, Bucuresti), E. Ceausu, C. P. Popescu, SA. Florescu, G. Târdei (Spitalul Clinic de Boli Infectioase si Tropicale Victor Babes, Bucuresti), C. Bejan, A. Teodor, G. Juganariu, C. Plesca, E. Duca (Spitalul Clinic de Boli Infectioase Sf. Parascheva, Iasi) France: N. Lenzi, Z. Lesieur [Inserm, F-CRIN, Innovative clinical research network in vaccinology (I-REIVAC)], P. Loulergue (CIC De Vaccinologie, Cochin-Pasteur Paris, I-REIVAC), F. Galtier (Hôpital Saint-Eloi, CHU de Montpellier, I-REIVAC), C. Agostini, M. Ray, C. Merle, V. Foulongne (CHU de Montpellier), B. Lina (Université Lyon 1, CNR Virus Influenza France Sud, Lyon), F. Lainé (Hôpital Pontchaillou, CHU de Rennes, I-REIVAC), S. De Guibert, G. Lagathu, P. Tattevin, S. Jouneau, A. Esvant, T. le Gallou (Hôpital Pontchaillou, CHU de Rennes), F. Carrat (UPMC Univ Paris 06, IPLESP UMRS 1136, Public health department, Hôpital Saint-Antoine, Paris), G. Mawuvi, F. CHAU (UMRS 1136, Paris). Finland: H. Nohynek, A. Haveri (National Institute for Health and Welfare) Lithuania: G. Gefenaite, D. Velyvyte (Department of Infectious Diseases of Lithuanian University of Health Sciences, Kaunas, Lithuania) L. Jancoriene, B. Zablockiene, A. Ambrozaitis (Clinic of Infectious, Chest Diseases, Dermatovenerology and Allergology, Vilnius University Faculty of Medicine, Vilnius, Lithuania; Centre of Infectious Diseases, Vilnius University Hospital Santaros Klinikos, Vilnius, Lithuania), R. Grimalauskaite, G. Damuleviciene, V. Lesauskaite (Department of Geriatrics, Lithuanian University of Health Sciences, Kaunas, Lithuania), A. Bagdonas (Department of Internal Medicine, Lithuanian University of Health Sciences) Portugal: B. Nunes, I. Kislaya, A.P. Rodrigues (National Health Institute Doutor Ricardo Jorge, Lisbon), V. Gomes, R. Côrte-Real (Centro Hospitalar de Lisboa Central, Lisbon), J. Poças, M.J. Peres (Centro Hospitalar de Setúbal, Setúbal) Croatia: Bernard K., S. Kurecic-Filipovic, V. Visekruna Vucina (Croatian Institute of Public Health), A. Topic, N. Papic, J. Budimir (University Hospital for Infectious Diseases “Dr.Fran Mihaljevic”) Hungary: B. Oroszi (Office of the Chief Medical Officer, Budapest) The Netherlands: A. Meijer, W. van der Hoek (National Institute for Public Health and the Environment (RIVM), Bilthoven), P.M. Schneeberger (Jeroen Bosch Hospital, 's Hertogenbosch)
